# Characterisation of tissue-type metabolic content in secondary progressive multiple sclerosis: a magnetic resonance spectroscopic imaging study

**DOI:** 10.1007/s00415-018-8903-y

**Published:** 2018-05-30

**Authors:** Ian Marshall, Michael J. Thrippleton, Mark E. Bastin, Daisy Mollison, David A. Dickie, Francesca M. Chappell, Scott I. K. Semple, Annette Cooper, Sue Pavitt, Gavin Giovannoni, Claudia A. M. Gandini Wheeler-Kingshott, Bhavana S. Solanky, Christopher J. Weir, Nigel Stallard, Clive Hawkins, Basil Sharrack, Jeremy Chataway, Peter Connick, Siddharthan Chandran, for the MS-SMART Trialists

**Affiliations:** 1 Centre for Clinical Brain Sciences, University of Edinburgh, Edinburgh, UK; 2 Institute of Cardiovascular and Medical Sciences, University of Glasgow, Glasgow, UK; 3 Centre for Cardiovascular Sciences, University of Edinburgh, Edinburgh, UK; 4 Edinburgh Imaging QMRI Facility, University of Edinburgh, Edinburgh, UK; 5 Dental Translational and Clinical Research Unit, School of Dentistry, Faculty of Medicine and Health, University of Leeds, Leeds, UK; 6 Department of Neurology, Barts and the London NHS Trust, London, UK; 7 UCL Institute of Neurology, Queen Square MS Centre, University College London, London, UK; 8 Department of Brain and Behavioural Sciences, University of Pavia, Pavia, Italy; 9 Brain MRI 3T Research Centre, IRCCS Mondino Foundation, Pavia, Italy; 10 Edinburgh Clinical Trials Unit, Usher Institute of Population Health Sciences and Informatics, University of Edinburgh, Edinburgh, UK; 11 Division of Health Sciences, University of Warwick, Warwick, UK; 12 Institute for Science and Technology in Medicine, Keele University, Newcastle, UK; 13 Academic Department of Neuroscience, The Sheffield NIHR Translational Neuroscience Biomedical Research Centre, University of Sheffield, Sheffield, UK; 14 Queen Square Multiple Sclerosis Centre, Department of Neuroinflammation, UCL Institute of Neurology, University College London, London, UK

**Keywords:** Multiple sclerosis, Magnetic resonance spectroscopy, Brain metabolites, White matter lesions, Normal-appearing white matter

## Abstract

Proton magnetic resonance spectroscopy yields metabolic information and has proved to be a useful addition to structural imaging in neurological diseases. We applied short-echo time Spectroscopic Imaging in a cohort of 42 patients with secondary progressive multiple sclerosis (SPMS). Linear modelling with respect to brain tissue type yielded metabolite levels that were significantly different in white matter lesions compared with normal-appearing white matter, suggestive of higher myelin turnover (higher choline), higher metabolic rate (higher creatine) and increased glial activity (higher myo-inositol) within the lesions. These findings suggest that the lesions have ongoing cellular activity that is not consistent with the usual assumption of ‘chronic’ lesions in SPMS, and may represent a target for repair therapies.

## Introduction

Multiple sclerosis (MS) is a disabling neurological disease affecting some 2.5 million people worldwide (http://www.mstrust.org.uk). There are currently no effective treatments for the progressive phases of MS when disability accumulates irreversibly. In clinical trials of potential disease-modifying therapies, the standard outcome measure is the Expanded Disability Status Scale (EDSS) [1]. In imaging studies, lesion load and brain atrophy are widely used biomarkers. White matter lesions (WML) are visible on routine magnetic resonance imaging (MRI) sequences, and represent current or previous inflammatory activity. More advanced MRI methods enable estimation of myelin status through white matter connectivity and magnetisation transfer imaging [2]. Complementary information on neuronal integrity is available from proton MR spectroscopy and spectroscopic imaging (MRSI) studies of brain metabolites.

A consistent finding of spectroscopic studies is that the level of the neuronal marker *N*-acetyl aspartate (NAA) is reduced in the normal-appearing white matter (NAWM) of patients compared with controls [3–5], and further reduced in WML [4, 6], although this latter finding may apply specifically to chronic rather than acute lesions [7]. In a longitudinal study, Obert et al. [8] found that NAA decreased in the NAWM of patients with secondary progressive MS (SPMS) and in the WML of patients with relapsing–remitting MS (RRMS).

Other findings from these studies are that choline, a potential marker of myelin turnover, is increased in acute lesions relative to NAWM [6], and in acute WML and NAWM relative to WM in control subjects [7]. Raised levels of myo-inositol, associated with glial activity, have been found in acute WML, chronic WML and NAWM relative to WM in controls [7], in grey matter (GM) and NAWM relative to controls [5], and in WML relative to NAWM [8]. Srinivasan et al. [7] found increased levels of glutamate, but not glutamine, in acute WML and NAWM relative to control WM.

The non-invasive nature of MRSI lends itself to use in longitudinal studies, both to monitor the natural progression of disease and in clinical trials of potential disease-modifying treatments. One such trial is MS-SMART (http://www.ms-smart.org), a 2-year, multicentre trial of three repurposed drugs for treatment of SPMS. Here, we report on MRSI measurements of brain metabolites made at the baseline time point of this trial. Using image segmentation and linear modelling we were able to investigate metabolite differences between tissue types.

## Methods

111 patients with SPMS who had had no relapses within the previous 3 months gave informed consent to be recruited in our centre as part of the study. At baseline, 43 patients (mean age 55 (standard deviation, SD 8) years; 30 female and mean EDSS score 6.0 (SD 0.7)) underwent MRSI as part of an MRI examination at 3T (Siemens Verio, Siemens Healthcare, Erlangen, Germany) using a standard 12-channel matrix head coil. PD-T2-weighted and FLAIR structural scans were acquired parallel to the anterior commissure–posterior commissure (ACPC) line with matrix 256 × 256, field of view (FOV) 250 × 250 mm and 60 contiguous slices 3 mm thick. The sequence timings were TR/TE1/TE2 = 3050/31/82 ms (turbo factor 7) and TR/TI/TE = 9500/2400/124 ms (turbo factor 28) respectively. A 3D inversion recovery prepared T1-weighted gradient echo scan (MPRAGE) was also acquired with matrix 256 × 256, field of view (FOV) 250 × 250 mm, 160 sagittal slices 1 mm thick, flip angle 8°, TR/TI/TE = 2400/1000/3 ms and parallel imaging acceleration factor 2.

**Fig. 1:**
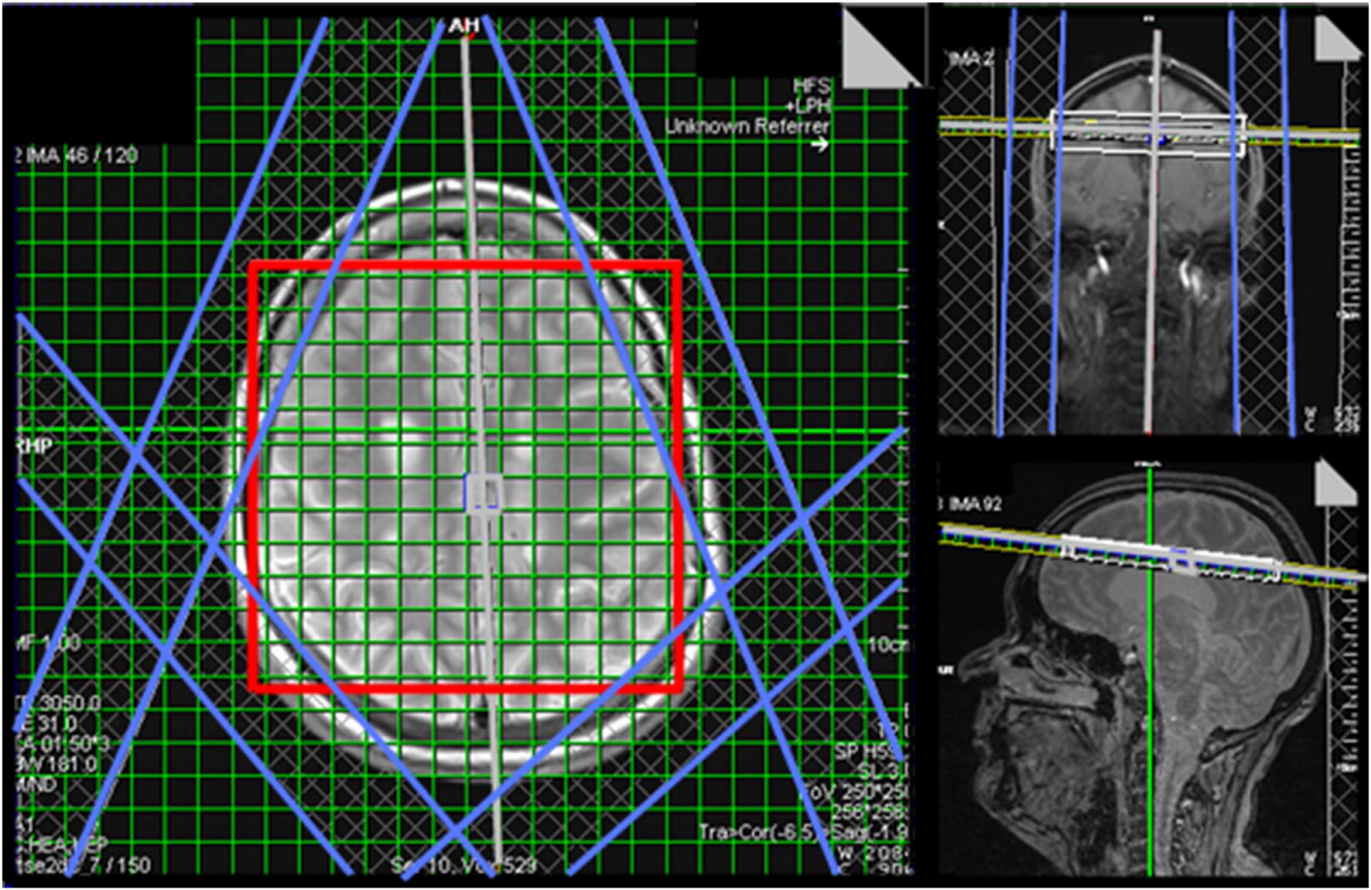
Prescription of the MRSI scan showing (left) the semi-LASER excitation region with red outline and outer volume suppression regions with blue outlines; (right) angulation of the MRSI slice parallel to the AC–PC line immediately above the lateral ventricles

Proton MRSI was carried out using a manufacturer-supplied prototype with semi-LASER excitation [9] and TR/TE of 2000/43 ms. Data were acquired from a single 10-mm-thick slice of spectroscopic voxels prescribed immediately above the lateral ventricles in the plane of the T2-weighted images (Fig. 1). Thus, the MRSI slice thickness extended through three adjacent T2-weighted images. The excitation volume was 120 mm × 120 mm. The spatial encoding matrix was 24 × 24 with elliptical *k*-space sampling, interpolated to 32 × 32 by the scanner, resulting in 1 mL spectroscopic voxels. Free induction decays consisted of 1024 samples with a dwell time of 500 µs. Weak water suppression was applied, together with four 30-mm-thick saturation bands positioned to suppress scalp lipid signals.

Spectroscopic data were analysed in LCModel [10] using a spectral basis set matching the semi-LASER sequence, generated using simulation routines within the FID-A toolkit [11]. The LCModel results for NAA, choline, creatine, myo-inositol and Glx (the sum of glutamate and glutamine) were multiplied by the scanner transmitter reference voltage to adjust for subject-specific loading of the head coil [12]. Finally, metabolite measurements were corrected for relaxation effects using literature values for T1, derived from NAWM in a group of RRMS and SPMS patients [7] to yield concentrations in Institutional Units.

Voxels were excluded from further analysis if they were judged to be not completely inside the brain or if the LCModel Cramer–Rao bounds (i.e., % standard deviations) of the fitted spectra exceeded 20% for any metabolite or 40% for myo-inositol [7]. Additionally, voxels judged by an experienced spectroscopist to have poor quality spectra (e.g., with grossly distorted baselines) were discarded.

The 3D T1-weighted and 2D FLAIR structural volumes were co-registered to the T2-weighted images and segmented to generate tissue probability maps for cerebrospinal fluid (CSF), GM, NAWM and WML using tools freely available in FSL (https://fsl.fmrib.ox.ac.uk) and ANTS [13]. WML probability masks were automatically created based on a FLAIR signal intensity threshold chosen empirically to provide WML masks that optimised the correspondence to masks manually segmented by an experienced neuroradiologist. The tissue segmentation maps were resampled to MRSI resolution and summed over three adjacent slices to match the MRSI slice thickness, thereby determining the overall fractional tissue content corresponding to each spectrum.

Tissue probabilities were used to predict metabolite concentrations (and ratios with respect to creatine) in a linear mixed model in SAS Studio 3.5 (http://www.sas.com). Subjects were regarded as random effects (i.e. a random intercept model) to allow for within-subject correlation of voxels and so that we could look specifically at *differences* in metabolite levels between tissue types within subject. To avoid problems with the collinearity of NAWM and GM, we used the variables (NAWM + GM), (NAWM-GM) and WML in the regression. Thus, the model was

$${C_{{\text{met}}}}={\text{ Intercept}}+{\beta _{\text{1}}}\left( {{\text{NAWM}}+{\text{GM}}} \right)+{\beta _{\text{2}}}\left( {{\text{NAWM}} - {\text{GM}}} \right)+{\beta _{\text{3}}}{\text{WML}}+\left( {{\text{subject effect}}} \right).$$

where the metabolite concentrations *C*_met_ and the tissue probabilities (NAWM, GM and WML) have been measured for each voxel. The *β* coefficients, Intercept and subject effects are estimated by the model. The model fit was assessed by examining plots of residuals. We also ran an extended model that included the EDSS score as a variable. 19(17) Patients had EDSS scores of 6.0(6.5), with the remaining six patients having scores in the range 4.0 to 5.5. Given that the data were so sparse for these latter values, we combined them with the patients having EDSS of 6.0, i.e., dichotomising the patients into two groups with EDSS ≤ 6.0 and EDSS = 6.5.

## Results

**Fig. 2:**
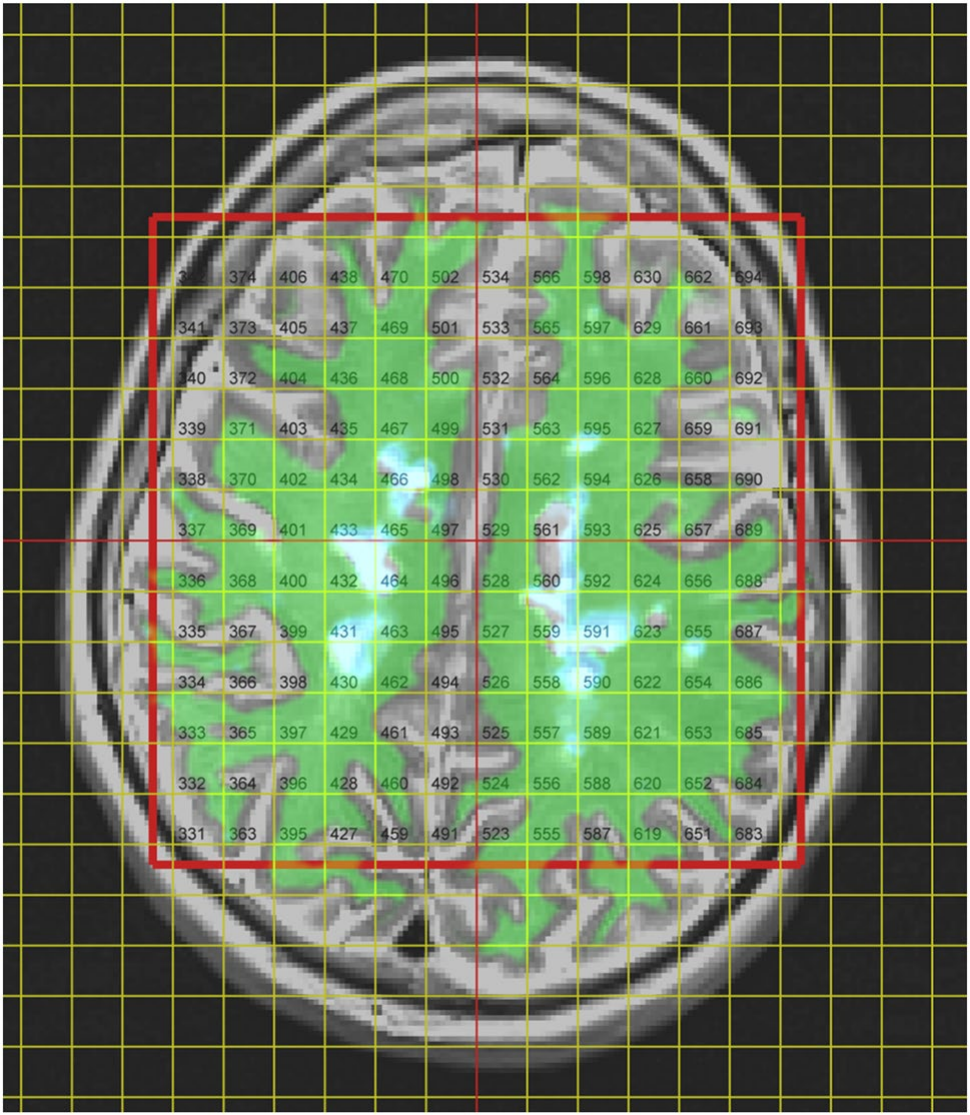
MRSI voxel grid and tissue segmentations overlaid on the T2-weighted image for a representative patient. The spectroscopic excitation volume is shown with a red outline. Green shading indicates NAWM and pale blue indicates WML

**Fig. 3:**
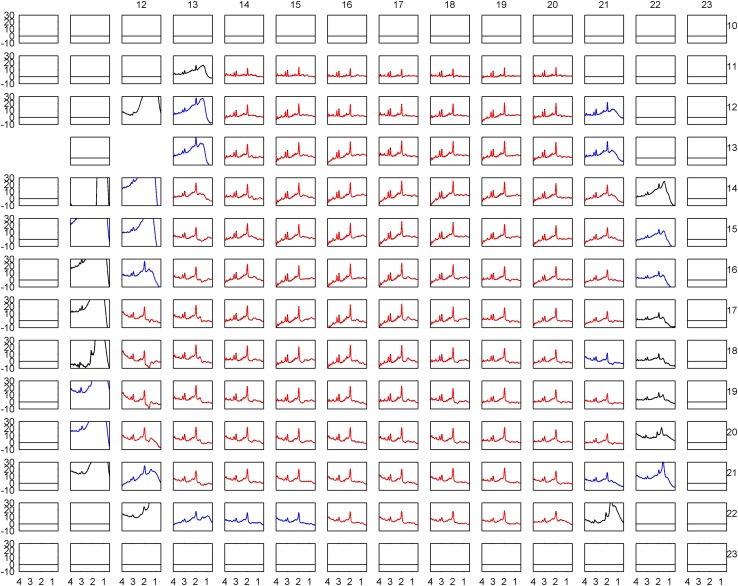
Spectra for the same patient as in Fig. 2 fitted by LCModel. Those shown in red passed the Cramer–Rao Bounds tests, whilst those shown in black did not. Spectra passing the CRB tests but judged to be of poor quality are shown in blue

**Fig. 4:**
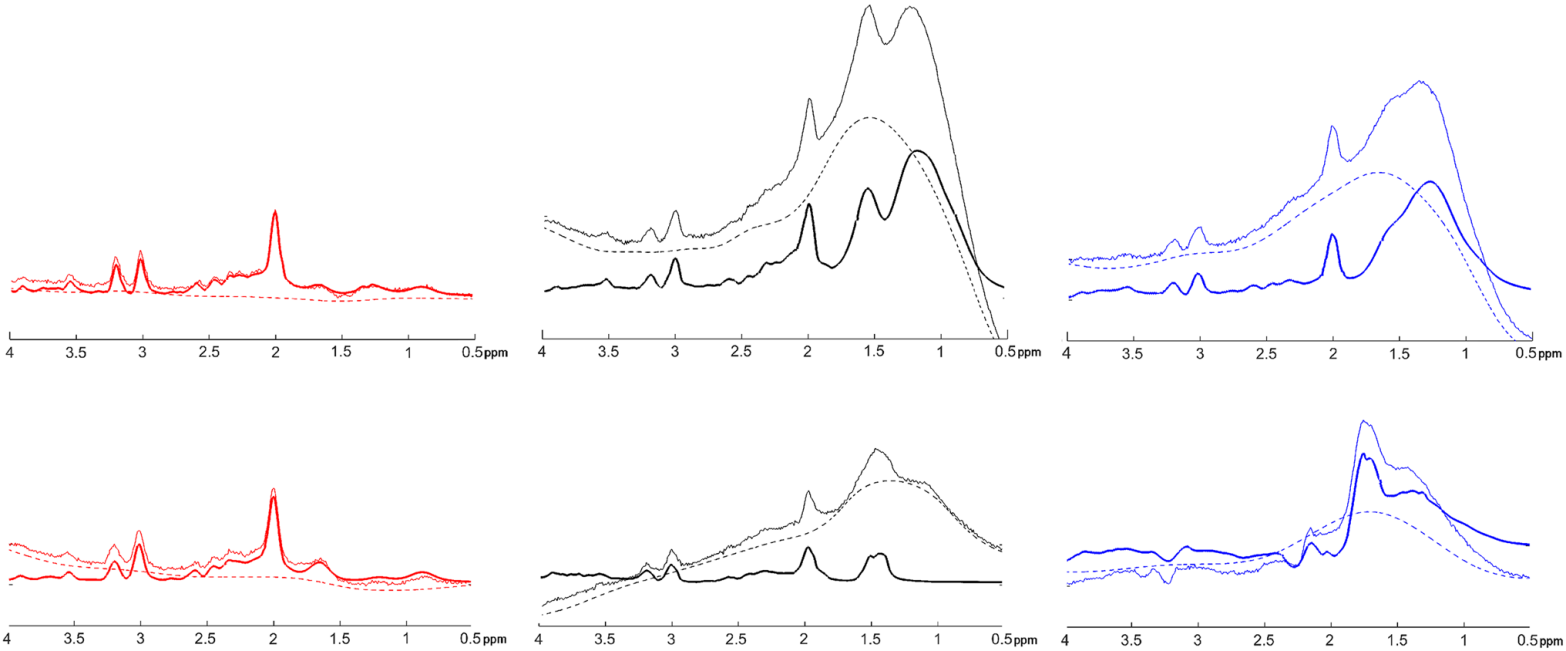
Representative examples of (left column, in red) spectra that passed the Cramer–Rao tests; (middle column, in black) spectra that failed the Cramer–Rao tests; (right column, in blue) spectra that passed the Cramer–Rao tests but which were rejected at visual inspection. LCModel baselines are shown with dashed lines, and spectral fits with heavy lines. All spectra have the same scaling

Image registration failed for one patient, who was removed from the analysis. In the remaining 42 patients, a total of 5349 spectroscopic voxels were located wholly within brain tissue, of which 4558 (85%) passed the Cramer–Rao tests. 916 (20%) of these voxels failed the visual test of spectral quality, leaving 3642 voxels (87 ± 17 per patient: range 33–116) in the final analysis. Tissue probabilities averaged across all accepted voxels were GM 31%, NAWM 56% and WML 3%, with the balance being CSF. An example of tissue segmentation is shown in Fig. 2. The overall mean (SD) linewidth of all accepted spectra was 7.0 (2.3) Hz. Representative fitted spectra are shown in Figs. 3 and 4.

**Fig. 5:**
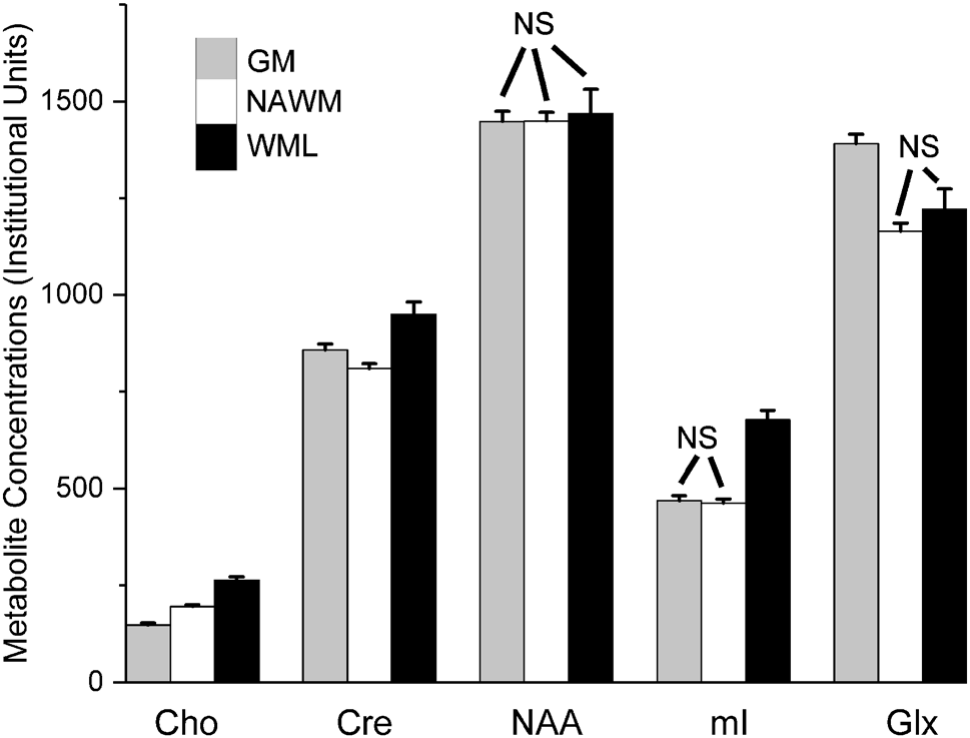
Principal metabolite concentrations (mean ± std error) in institutional units with respect to brain tissue type. Columns are shaded grey, white and black to indicate GM, NAWM and WML, respectively. NS: not significantly different. Further details in Table 1

**Fig. 6:**
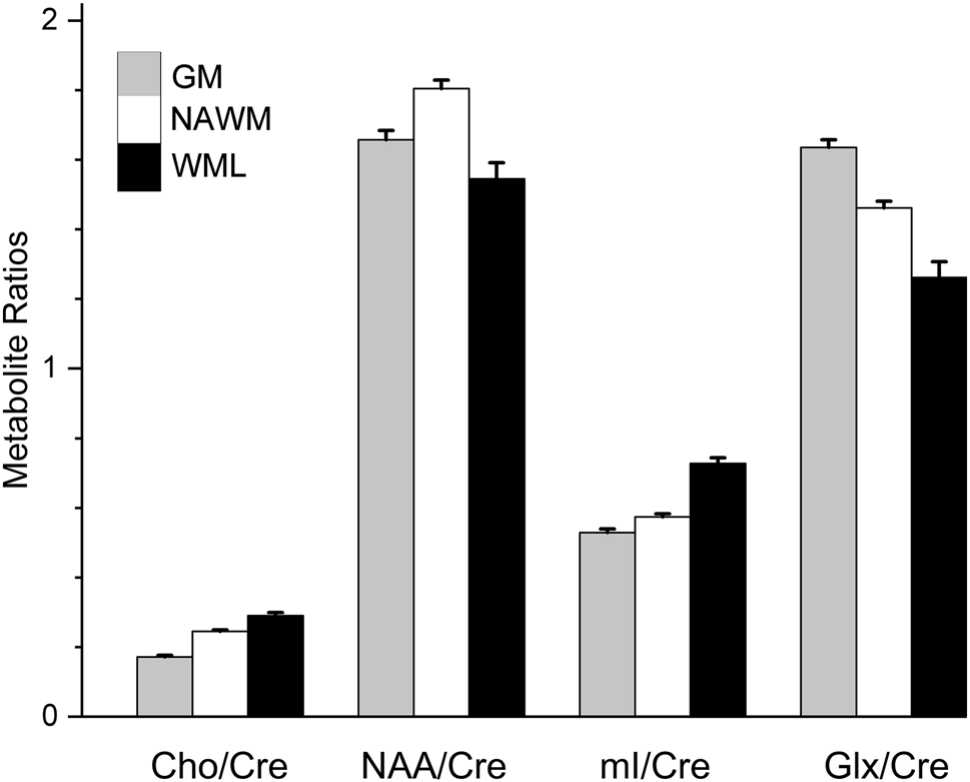
Principal metabolite ratios (mean ± std error) relative to creatine with respect to brain tissue type. Columns are shaded grey, white and black to indicate GM, NAWM and WML, respectively. Further details in Table 2

Tissue-specific metabolite concentrations determined from the linear mixed model (disregarding EDSS score) are given in Table 1 and Fig. 5. Several previous studies [3, 6, 14, 15] have reported ratios with respect to creatine and we have included these in Table 2 and Fig. 6. All metabolite concentrations and ratios are reported as mean (standard error).

**Table 1:** Principal metabolite concentrations in institutional units (mean ± std error) with respect to brain tissue type in 42 patients with SPMS

	NAWM	WML	GM
Choline	* 196 ± 5	263 ± 9 *	* 147 ± 5
Creatine	* 809 ± 14	958 ± 32 *	* 858 ± 15
NAA	***0.74*** 1449 ± 23	1469 ± 62 ***0.96***	***0.71*** 1447 ± 27
Myo-inositol	* 463 ± 10	677 ± 24 ***0.50***	* 469 ± 13
Glx	***0.28*** 1164 ± 21	1220 ± 53 *	* 1390 ± 24

Significance of pairwise comparisons is shown as actual *p* values or as * (*p* < 0.01)

*NAWM* normal-appearing white matter, *WML* white matter lesions, *GM* grey matter

**Table 2:** Principal metabolite ratios relative to creatine (mean ± std error) with respect to brain tissue type in 42 patients with SPMS

	NAWM	WML	GM
Choline/creatine	0.25 ± 0.01	0.29 ± 0.01	0.17 ± 0.01
NAA/creatine	1.80 ± 0.02	1.55 ± 0.05	1.66 ± 0.03
Myo-inositol/creatine	0.57 ± 0.01	0.73 ± 0.02	0.53 ± 0.01
Glx/creatine	1.46 ± 0.02	1.26 ± 0.05	1.64 ± 0.02

All pairwise comparisons are statistically significant at *p* < 0.01

*NAWM* normal-appearing white matter, *WML* white matter lesions, *GM* grey matter

We found that the level of choline was higher in NAWM than GM (196 (5) vs 147 (5) Institutional Units) whereas creatine was lower (809 (14) vs 858 (15)) as was Glx (1164 (21) vs 1392 (24)) (p < 0.001 for all these comparisons). There were no significant differences in myo-inositol level between GM and NAWM, nor in NAA levels between any of GM, NAWM and lesions.

Choline, creatine and myo-inositol levels were all significantly (*p* < 0.001) higher in WML compared with NAWM and GM. There were no significant differences in NAA or Glx levels between WML and NAWM, but Glx was lower in WML than in GM (*p* = 0.002).

Significant differences were found between all three tissue types for all the metabolite ratios with respect to creatine. Specifically, despite the higher creatine in WML compared with NAWM (Table 1), the ratios choline/creatine and myo-inositol/creatine are also higher in WML than NAWM. On the other hand, the ratios NAA/creatine and Glx/creatine are lower in WML than NAWM, whereas NAA and Glx were individually not different between WML and NAWM.

In the ancillary mixed model with dichotomised EDSS scores, we found no significant differences with respect to EDSS for any metabolite or metabolite ratio (all *p* values > 0.3).

## Discussion

Short-TE MRSI coupled with tissue segmentation and linear modelling enabled estimation of brain metabolite levels in GM, NAWM and WML despite very few voxels having ‘pure’ tissue content. Including subjects as random effects enabled us to focus on metabolite *differences* between tissue types. Our findings for NAWM compared with GM are consistent with the literature except that we found no significant difference in myo-inositol levels. Of particular interest is our finding that WML had higher choline, creatine and myo-inositol levels than did NAWM, suggesting ongoing abnormal metabolism in these lesions.

### NAWM–GM differences

Our findings for the concentrations of choline, creatine and Glx in NAWM compared with GM are consistent with previous studies in healthy controls [12, 16, 17].

We found no significant difference in NAA level between NAWM and GM. Studies in control subjects have found NAA to be either lower or higher in GM than in WM, with a range from 15% lower to 46% higher being reported by Schuff et al. [16]. They speculated that these mixed findings might in part be due to regional variations, the age of the participants, relaxation values or technical differences in acquisition.

Our finding of no significant difference between NAWM and GM levels of myo-inositol differs from Llufriu et al. [5] who found levels 50% higher in GM in a cohort of 59 patients with MS of undisclosed subtype. Similarly, in an early single-voxel MRS study of healthy young controls, Michaelis et al. [12] found myo-inositol levels 30% higher in GM than WM. The different excitation scheme (STEAM rather than the usual PRESS or semi-LASER), shorter echo time (20 vs 43 ms) and mixed tissue content in that study may partly explain the disparate findings.

### WML–NAWM differences

Numerous studies have compared NAWM in MS patients with NAWM in control subjects, but relatively few have compared NAWM with WML. This is likely due to the difficulties of making MRS measurements in lesions of generally small size, irregular shape and low overall load. Cucurella et al. [4] used a single 8 mL MRS voxel in each of 18 SPMS patients, placed either in predominately NAWM or predominately WML, finding that NAA was lower in WML than NAWM. Kapeller et al. [18] found reduced NAA and increased myo-inositol in WML relative to NAWM in a group of 32 patients with MS of undisclosed subtype.

Using a mixed linear model we found no difference in NAA or Glx between NAWM and WML although the ratios NAA/cre and Glx/cre were respectively lower and higher in WML. As did Obert et al. [8], we found increased myo-inositol in WML relative to NAWM.

Srinivasan et al. [7] used a custom single-voxel spectroscopic sequence designed to separate the heavily overlapped glutamate and glutamine components of the composite ‘Glx’ signal. In a mixed cohort of RRMS, primary and secondary progressive MS patients, they compared 12 chronic WML voxels with 17 NAWM voxels. They found that NAA, choline and glutamate were lower in chronic WML than NAWM. When including the signal from glutamine, there was no difference for the combined Glx, as we found in the present study.

Our finding of significantly higher levels of choline, creatine and myo-inositol in WML relative to NAWM suggest higher membrane turnover, higher metabolic rate and increased glial activity, respectively. Inflammatory disease activity was low in this cohort, as evidenced by the eligibility criteria and the structural MR brain features. The cohort can, therefore, reasonably be expected to have WML that were overwhelmingly non-recent. However, it would be simplistic to assume that the lesions were “chronic” (a neuropathological descriptor defined by apparent biological inertness at post mortem examination) despite the phenotypic definition of “secondary progressive MS”. Indeed, our findings suggest that non-recent WML in SPMS patients who are EDSS ≤ 6.5 (i.e., not EDSS 10 [dead]) exhibit ongoing cellular activity that is not consistent with the pathological definition of “chronic”, and may represent a targetable substrate for repair therapies.

### General points

A strength of our study is that the study group comprised a well-characterised cohort of SPMS patients with a narrow range of EDSS scores. A limitation is that there was no healthy control group and so it is difficult to know how ‘normal’ the NAWM really is at this stage of the disease. The MS-SMART study is designed to compare two-year progression in four randomised groups (three candidate drug treatments and one placebo) and the main analysis will be comparison of clinical status, structural features and metabolites for each of the candidate drugs relative to placebo at the end of the trial. Interestingly, Obert et al. [8] have recently reported a reduction in NAWM NAA in 15 SPMS patients over the course of a 2-year study. Our finding that metabolite values and ratios were not related to the EDSS scores at baseline in our study group is not surprising given the study design.

We used a single slice of MRSI, nominally placed immediately above the lateral ventricles. Depending on the exact placement of the slice and the size and shape of the patient’s brain, the potential number of brain voxels varied. However, reduced numbers were mainly due to rejection based on the Cramer Rao bounds and visual quality assessment. The latter is time consuming and necessarily subjective, thus leading to interest in developing machine-learning approaches to spectral quality control [19].

We corrected the ‘raw’ metabolite measurements using assumed T1 relaxation times and for loading of the coil, resulting in institutional units. Other studies [3, 6, 15] have avoided these steps and reported ratios with respect to creatine, which was found or assumed to have a similar concentration in different tissues. In fact, creatine is often assumed to be a stable benchmark for hypothesis testing in MRS studies. Our work suggests that this is not necessarily the case, since taking ratios with respect to creatine changed our findings for NAA and Glx. We, therefore, advise caution for any MRS analysis based on the assumption of creatine being a stable reference.

The current analysis takes no account of the position of the voxels within the MRSI slice, and thus effectively averages tissue metabolite concentration values across the whole slice. Future methodological refinements could include regional analysis and absolute quantification of metabolites. Although the latter requires the use of calibrated phantoms and is time consuming, interpreting the measurements in terms of absolute concentrations (e.g., millimoles per litre) rather than Institutional Units should make it easier to compare results between centres. The complex nature of MRS/MRSI studies requires the detailed reporting of acquisition and analysis methods.

## Conclusion

MS-SMART is one of the largest studies of SPMS, with 440 patients recruited across 13 UK centres. Longitudinal changes in clinical status, structural features and metabolites will be investigated at the 2-year endpoint. Preliminary findings at baseline in our MRSI sub-group show higher choline, creatine and myo-inositol in WML compared with NAWM, indicating higher myelin turnover, higher metabolic rate and increased glial activity respectively. These suggest that the lesions may have continuing, abnormal metabolism despite these patients being in a progressive phase of their disease during which lesions are often assumed to be ‘chronic’ and not active. This finding may have wider implications for the understanding of pathobiology of non-recent lesions in vivo, and for stratification for studies that evaluate repair therapies. Adequately powered longitudinal studies are necessary to establish the usefulness of MRSI and other quantitative MRI techniques in monitoring disease progression and evaluating potential treatments.
